# Tobramycin Systemic Absorption in Lung Transplant Recipients Treated With Inhaled Tobramycin: A Cohort Study

**DOI:** 10.3389/ti.2024.12579

**Published:** 2024-03-28

**Authors:** Abiu Sempere, Ibai Los-Arcos, Judith Sacanell, Cristina Berastegui, David Campany-Herrero, Jaume Vima, María Teresa Martín-Gómez, Leire Sánchez, Daniel Martínez-González, Carles Bravo, Oscar Len, Joan Gavaldà

**Affiliations:** ^1^ Infectious Diseases Department, Hospital Universitari Vall d’Hebron, Barcelona, Spain; ^2^ Critical Care Department, Hospital Universitari Vall d’Hebron, Barcelona, Spain; ^3^ Lung Transplant Unit, Hospital Universitari Vall d’Hebron, Barcelona, Spain; ^4^ CIBER de Enfermedades Respiratorias (CIBERES), Barcelona, Spain; ^5^ Pharmacy Department, Hospital Universitari Vall d’Hebron, Barcelona, Spain; ^6^ Department of Clinical Biochemistry, Central Clinical Laboratories, Hospital Universitari Vall d’Hebron, Barcelona, Spain; ^7^ Microbiology Department, Hospital Universitari Vall d’Hebron, Barcelona, Spain; ^8^ Thoracic Surgery Department, Hospital Universitari Vall d’Hebron, Barcelona, Spain; ^9^ CIBERINFEC, ISCIII—CIBER de Enfermedades Infecciosas, Instituto de Salud Carlos III, Madrid, Spain

**Keywords:** aminoglycosides, drug levels, nebulized, acute kidney injury, nephrotoxicity

## Abstract

Inhaled tobramycin treatment has been associated with nephrotoxicity in some case reports, but limited data are available about serum levels and its possible systemic absorption in lung transplant recipients (LTR). We conducted a single-center, observational and retrospective study of all adult (>18 years old) LTR treated with inhaled tobramycin for at least 3 days between June 2019 and February 2022. Trough serum levels were collected and >2 μg/mL was considered a high drug level. The primary outcome assessed the presence of detectable trough levels, while the secondary outcome focused on the occurrence of acute kidney injury (AKI) in individuals with detectable trough levels. Thirty-four patients, with a median age of 60 years, were enrolled. The primary indications for treatment were donor bronchial aspirate bacterial isolation (18 patients) and tracheobronchitis (15 patients). In total, 28 patients (82%) exhibited detectable serum levels, with 9 (26%) presenting high levels (>2 μg/mL). Furthermore, 9 patients (26%) developed acute kidney injury during the treatment course. Median trough tobramycin levels were significantly elevated in invasively mechanically ventilated patients compared to non-ventilated individuals (2.5 μg/mL vs. 0.48 μg/mL) (*p* < 0.001). Inhaled tobramycin administration in LTRs, particularly in those requiring invasive mechanical ventilation, may result in substantial systemic absorption.

## Graphical Abstract

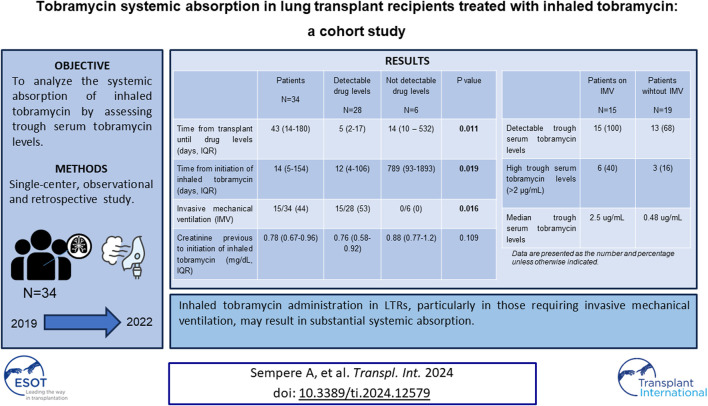

## Introduction

Bacteria are the most frequent cause of infection in lung transplant recipients (LTR) [1], leading to tracheobronchitis or pneumonia and might affect the bronchial suture [2]. To control such infections, inhaled antibiotics are frequently used, although few published data are available [1]. We have previously described the use of inhaled antibiotics to prevent donor-derived infection, even in multidrug resistant bacteria [3]. The efficacy of secondary prophylaxis involving nebulized antibiotics during the intensive care unit (ICU) admission of lung transplant recipients has also been documented [4].

The inhalational route facilitates the direct delivery of high antibiotic concentrations to the lungs, exposing bacteria to lethal concentrations while minimizing potential systemic toxicity by limiting absorption [5]. Colistin and tobramycin are very common nebulized antibiotics. Several studies have indicated that plasma levels in patients treated with nebulized colistin for ventilator-associated pneumonia are either undetectable or very low, falling below levels associated with potential nephrotoxicity [6]. Systemic tobramycin is recognized for its adverse effects, encompassing nephrotoxicity and ototoxicity [7]. Consequently, it becomes crucial to ascertain the extent of systemic absorption of inhaled, particularly in LTR who frequently receive other nephrotoxic drugs such as calcineurin inhibitors [8]. However, there are few studies describing the systemic absorption of inhaled tobramycin. Detectable tobramycin levels have been reported, mainly in patients with cystic fibrosis and associated with nephrotoxicity in some cases [9–11]. Nevertheless, these findings have not been confirmed in other studies [12–14]. In the setting of lung transplantation, cases of drug absorption and nephrotoxicity have also been documented [15–19].

The aim of our study is to analyze the systemic absorption of inhaled tobramycin by assessing trough serum tobramycin levels in a cohort of LTR treated with inhaled tobramycin.

## Material and Methods

### Patients and Setting

This is an observational retrospective study performed at Hospital Universitari Vall d’Hebron, a 1,000-bed teaching hospital in Barcelona (Spain). Our institution is the leading lung transplant center in Spain, conducting approximately 120 lung transplants annually. The study encompassed all consecutive adult patients (≥18 years of age) who underwent lung transplantation and received inhaled tobramycin treatment for a duration of at least 3 days. The study period extended from June 2019 to February 2022.

Following a clinical protocol implemented by the lung transplant unit since June 2019, tobramycin trough levels were systematically assessed in all lung transplant recipients undergoing nebulized tobramycin treatment. The frequency of drug monitoring was determined by the treating physician according to usual clinical practice.

The Clinical Research Ethics Committee of our hospital approved the study (EOM(AG)021/2022(5980)) and waived the requirement for informed consent.

### Lung Transplant Antibiotic Protocol

The preventive antibiotic strategy in our center involves the administration of intravenous amoxicillin-clavulanate and ceftazidime during the surgical lung transplant procedure. This intravenous regimen is continued until the results of perioperative cultures are obtained. In cases where intraoperative bacterial isolation is identified in either the recipient or donor, intravenous antibiotics are customized based on antibiotic susceptibility patterns and extended for a duration of 10–14 days. Furthermore, inhaled antibiotics such as tobramycin (300 mg every 12 h) or colistin (2–5 million units every 8 h) are introduced, again guided by the antibiotic susceptibility profile. Tobramycin was mainly used when bacterial isolates were resistant to colistin. This inhaled antibiotic regimen is typically continued for a period of 2–4 months. The nebulized antibiotic is maintained on an outpatient basis assessing the risk-benefit ratio according to the criteria established by the treating physician.

### Data Collection

Patients were identified through the pharmacy database. Demographic, clinical, and microbiological data were collected from electronic medical records and entered anonymously into a database, specifically created for the study.

### Definitions

Respiratory tract infections were defined as outlined by the multidisciplinary working group of The International Society for Heart and Lung Transplantation [2]. Donor lung bacterial isolation was based on the isolation of any amount of bacteria in a selected and protected bronchial aspirate performed after opening the bronchial suture just prior the implantation.

Acute kidney injury (AKI) was defined as a reduction in renal function within 48 h characterized by an absolute increase in the serum creatinine level exceeding 0.3 mg/dL or a 50% increase above the baseline value [20].

Tobramycin was prescribed at the discretion of the treating physician and administered over 30 min. Treatment was nebulized via Aeroneb Pro in mechanically ventilated patients and via vibrating mesh-nebulizer nebulizer in the other patients. The prescribed dosage was 300 mg/5 mL (TOBI^®^) every 12 h and the treating physician determined the duration of treatment. To monitor tobramycin serum levels, blood samples were collected 30 min before each dosing to ensure measurement at trough concentration. We considered high tobramycin serum levels those concentrations exceeding 2 μg/mL [21, 22].

The primary outcome was the presence of detectable trough serum levels of tobramycin and high tobramycin levels (>2 μg/mL). The secondary outcome was to describe the presence of acute kidney injury in patients with detectable trough serum levels of tobramycin.

Tobramycin drug levels were measured in serum with homogeneus particle-enhanced turbidimetric immunoassay (QMS-ThermoFisher) and laboratory lower limit of detection was 0.1 μg/mL.

### Statistical Analysis

Categorical values were expressed in absolute numbers and percentages, while quantitative variables were reported as medians and interquartile ranges (IQRs). Wilcoxon rank sum test was used to compare quantitative variables and Fisher exact test to compare categorical variables. Undetectable (<0.1 μg/mL) drug levels were computed as 0, and in patients with more than one detectable drug level the higher drug level was selected for the analysis.

## Results

A total of 34 patients, with a median age of 60 years, were enrolled in the study. Most patients, 31 (91%), underwent a bilateral lung transplantation. Baseline characteristics are summarized in Table 1. All patients except one received a tacrolimus-based immunosuppressive treatment.

**TABLE 1:** Comparison of demographic variables between patients with detectable tobramycin levels vs. patients with undetectable levels.

	Patients (*N* = 34)	Detectable drug levels (*N* = 28)	Not detectable drug levels (*N* = 6)	*p*-value
Male sex	21 (62)	18 (86)	3 (50)	0.848
Age, years, median (IQR)	60 (52–65)	60 (52–64)	62 (51–66)	0.421
Lung disease				0.283
Pulmonary fibrosis	13 (38)	11 (39)	2 (33)	
Chronic obstructive pulmonary disease	12 (35)	10 (36)	2 (33)	
Bronchiolitis obliterans	2 (6)	2 (7)	0	
Bronchiectasis	1 (3)	1 (3.5)	0	
COVID-19 pneumonia	1 (3)	1 (3.5)	0	
Cystic fibrosis	1 (3)	0	1 (17)	
Pleuropulmonary fibroelastosis	1 (3)	0	1 (17)	
Pulmonary alveolar proteinosis	1 (3)	1 (3.5)	0	
Pulmonary lymphangioleiomyomatosis	1 (3)	1 (3.5)	0	
Pulmonary veno-occlusive disease	1 (3)	1 (3.5)	0	
Type of lung transplant				
Single	3 (9)	0	3 (50)	—
Bilateral	31 (91)	28 (100)	3 (50)	0.349

Data are presented as the number and percentage unless otherwise indicated.

Inhaled tobramycin was primarily initiated by bacterial isolation in donor bronchial aspirate (*n* = 18), tracheobronchitis (*n* = 15), pneumonia (*n* = 4) and bronchial suture infection (*n* = 1). Four patients presented both donor bronchial aspirate bacterial isolation and lower respiratory tract infection. The main isolated bacteria were *Staphylococcus aureus* (*n* = 17), Enterobacterales (*n* = 14) and *Pseudomonas aeruginosa* (*n* = 5). Additional microbiological data as well as main variables for all patients are provided in Supplementary Tables S1, S2.

Twenty-nine patients received tobramycin at a dose of 300 mg/12h, and an additional 5 LTR patients were administered 300 mg/24 h (adjusted by the lung transplant physician in the outpatient setting). No patients received intravenous tobramycin, other intravenous aminoglycosides, intravenous colistin, vancomycin or other nephrotoxic agents concurrently, other than calcineurin inhibitors.

Tobramycin trough levels were determined at least twice in 18 patients (53%). Tobramycin was detected at least once in 28 patients (82%), with a median value of 0.76 μg/mL (IQR 0.38–2.2). Nine patients (26%) presented high tobramycin levels, with a median value of 3.81 μg/mL (IQR 2.39–6.65). All patients on IMV (*n* = 15) had detectable serum tobramycin levels after a median of 5 days (IQR 4–9) of inhaled treatment.

The median creatinine value before initiating nebulized tobramycin was 0.78 mg/dL (IQR 0.67–0.96) and two patients (5%) had a history of previous renal failure.

Inhaled tobramycin treatment was discontinued in 11 patients (32%) due to either high drug levels or acute kidney injury. Nine patients developed AKI after a median of 28 days (IQR 4–125) of nebulized tobramycin, with a median peak creatinine of 1.8 mg/dL (IQR 1.6–2.1). The median drug level in these patients was 2.8 μg/mL (IQR 1.9–6.3). Three of these patients did not recover their baseline renal function by 6-month.

Variables among patients with detectable and undetectable tobramycin levels are compared in Tables 1, 2. Patients with detectable levels exhibited a shorter time from both transplantation (12 vs. 789 days, *p* = 0.019) and antibiotic initiation until drug levels (5 vs. 14 days, *p* = 0.011). Additionally, patients with detectable levels were more frequently subjected to invasive mechanical ventilation (53% vs. 0, *p* = 0.016) Moreover, the median trough drug levels were significantly higher in invasively mechanically ventilated patients compared to those not ventilated (2.5 μg/mL vs. 0.48 μg/mL) (*p* < 0.001).

**TABLE 2:** Comparison of main variables between patients with detectable tobramycin levels vs. patients with undetectable levels.

	Patients *N* = 34	Detectable drug levels *N* = 28	Not detectable drug levels *N* = 6	*p*-value
Time from transplant until drug levels (days, IQR)	43 (14–180)	5 (2–17)	14 (10–532)	**0.011**
Time from initiation of inhaled tobramycin (days, IQR)	14 (5–154)	12 (4–106)	789 (93–1893)	**0.019**
Invasive mechanical ventilation	15/34 (44)	15/28 (53)	0/6 (0)	**0.016**
Creatinine previous to initiation of inhaled tobramycin (mg/dL, IQR)	0.78 (0.67–0.96)	0.76 (0.58–0.92)	0.88 (0.77–1.2)	0.109
Calcineurin inhibitor trough levels previous to initiation of inhaled tobramycin (mg/dL, IQR)	9.4 (8.1–12.1)	9.8 (8.1–12.8)	8.9 (8–10.1)	0.634
Calcineurin inhibitor first trough levels after initiation of inhaled tobramycin	8.4 (6.4–12.1)	8.2 (7.7–11.1)	12.1 (6.6–13.5)	0.594
Hypoalbuminemia previous to initiation of inhaled tobramycin	23/34 (67)	22/28 (78)	1/6 (17)	0.0921
Hypoalbuminemia at first measure after initiation of inhaled tobramycin	18/34	18/28	0/6	0.260

Data are presented as the number and percentage unless otherwise indicated.

Bold values indicate p < 0.05.

## Discussion

In our study, 82% of LTR exhibited detectable tobramycin drug levels, with 26% of patients demonstrating high tobramycin levels and 26% developing acute kidney injury. Notably, all recently transplanted patients on mechanical ventilation had detectable tobramycin levels.

A prior retrospective study, including both amikacin and tobramycin treatments, reported 39% detectable drug levels in LTR [18]. In a multivariate analysis from that study, factors such as cystic fibrosis, lung transplantation, chronic kidney disease, mechanical ventilation, and use of tobramycin instead of amikacin were associated with detectable drug levels. In our study, mechanical ventilation was also associated with higher trough drug levels in LTR. Additionally, in a recent study involving non-transplant patients on mechanical ventilation treated with inhaled tobramycin, 66% of patients had detectable drug levels [23]. Our study results also suggest that IMV, could promote the systemic absorption of tobramycin. Variables such as the time from transplantation until performing drug levels or the time from initiation of nebulized tobramycin are likely influenced by invasive mechanical ventilation. The systemic absorption of inhaled tobramycin during mechanical ventilation could be attributed to improved aerosol delivery, changes in pulmonary physiology, and increased vascular permeability due to lung tissue damage [18, 23]. These two studies [18, 23] did not demonstrate a statistically significant association between detectable levels of tobramycin and acute renal failure. However, in one of the studies, the levels were not analyzed as a continuous variable, and in both cases, the drug levels were not trough drug levels. Some reported cases of suspected nephrotoxicity due to inhaled tobramycin systemic absorption have involved non-mechanically ventilated LTR [15–17]. In these cases, high trough tobramycin levels were detected (8.7 mg/mL [16] and 2.7 mg/mL [17]), with recovery of previous renal function observed after discontinuation of inhaled tobramycin.

In our study, nine patients (26%) with detectable levels presented with renal failure. Of interest, six out nine patients who experienced AKI recovered their baseline renal function, while three (33%) did not. LTR possess numerous risk factors for nephrotoxicity, including immunosuppressive drugs (mainly calcineurin inhibitors), haemodynamic instability, exposure to other nephrotoxic drugs, or diabetes mellitus, among others. Consequently, the etiology of renal failure in these patients is often multifactorial and challenging to discern. AKI is highly prevalent after lung transplantation (39%–62%) and is associated with increased mortality [24]. Given the need of calcineurin inhibitors, minimizing exposure to other nephrotoxic agents is recommended [8]. Elevated trough levels of systemic aminoglycosides are correlated with a heightened risk of nephrotoxicity [7]. Therefore, the potential for systemic absorption of inhaled tobramycin in lung transplant recipients should be considered. Vestibular toxicity has also been reported in some cases [16], with one suspected case in our cohort that might have gone undetected.

Several limitations characterize our study, including its retrospective nature, limited sample size, and variability in the timing of tobramycin level measurements according to daily clinical practice. Furthermore, different doses of tobramycin (300 mg every 24 h) were administered to some patients. Nevertheless, our study provides insights into a cohort of lung transplant recipients undergoing nebulized tobramycin treatment at various stages of lung transplantation.

In conclusion, our findings suggest that inhaled tobramycin in LTR, particularly those on invasive mechanical ventilation, may undergo significant systemic absorption. Monitoring of tobramycin trough levels seems advisable to mitigate potential drug absorption.

## Data Availability

The original contributions presented in the study are included in the article/Supplementary Material, further inquiries can be directed to the corresponding author.

## References

[1] Cervera C van Delden C Gavaldà J Welte T Akova M Carratalà J Multidrug-Resistant Bacteria in Solid Organ Transplant Recipients. Clin Microbiol Infect (2014) 20(7):49–73. 10.1111/1469-0691.12687 24861521

[2] Husain S Mooney ML Danziger-Isakov L Mattner F Singh N Avery R A 2010 Working Formulation for the Standardization of Definitions of Infections in Cardiothoracic Transplant Recipients. J Heart Lung Transpl (2011) 30(4):361–74. 10.1016/j.healun.2011.01.701 PMC7172457 21419994

[3] Bunsow E Los-Arcos I Martin-Gómez MT Bello I Pont T Berastegui C Donor-Derived Bacterial Infections in Lung Transplant Recipients in the Era of Multidrug Resistance. J Infect (2020) 80(2):190–6. 10.1016/j.jinf.2019.12.006 31843689

[4] Tran-Dinh A Slassi L De Tymowski C Assadi M Tanaka S Zappella N Secondary Prophylaxis With Inhaled Colistin to Prevent Recurrence of *Pseudomonas Aeruginosa* and Extended-Spectrum β-Lactamase-Producing Enterobacterales Pneumonia in ICU After Lung Transplantation: A Before-And-After Retrospective Cohort Analysis. Transplantation (2022) 106(11):2232–40. 10.1097/TP.0000000000004187 35675449

[5] Wood GC. Aerosolized Antibiotics for Treating Hospital-Acquired and Ventilator-Associated Pneumonia. Expert Rev Anti Infect Ther (2011) 9(11):993–1000. 10.1586/eri.11.126 22029519

[6] Karaiskos I Gkoufa A Polyzou E Schinas G Athanassa Z Akinosoglou K. High-Dose Nebulized Colistin Methanesulfonate and the Role in Hospital-Acquired Pneumonia Caused by Gram-Negative Bacteria With Difficult-To-Treat Resistance: A Review. Microorganisms (2023) 11(6):1459. 10.3390/microorganisms11061459 37374959 PMC10304325

[7] Destache CJ. Aminoglycoside-Induced Nephrotoxicity--A Focus on Monitoring: A Review of Literature. J Pharm Pract (2014) 27(6):562–6. 10.1177/0897190014546102 25124375

[8] Ahya VN Diamond JM. Lung Transplantation. Med Clin North Am (2019) 103(3):425–33. 10.1016/j.mcna.2018.12.003 30955511

[9] Guy EL Bosomworth M Denton M Conway SP Brownlee KG Lee TW. Serum Tobramycin Levels Following Delivery of Tobramycin (Tobi) Via eFlow Advanced Nebuliser in Children With Cystic Fibrosis. J Cyst Fibros (2010) 9(4):292–5. 10.1016/j.jcf.2010.03.007 20427245

[10] Miller T Pastuch C Garavaglia L Gannon K Parravani A. Unknown Renal Impairment: A Rare Case of Inhaled Tobramycin Induced Acute Kidney Injury in a Cystic Fibrosis Patient. Antibiotics (Basel) (2021) 10(4):424. 10.3390/antibiotics10040424 33921466 PMC8070657

[11] Cannella CA Wilkinson ST. Acute Renal Failure Associated With Inhaled Tobramycin. Am J Health Syst Pharm (2006) 63(19):1858–61. 10.2146/ajhp060196 16990632

[12] van Koningsbruggen-Rietschel S Heuer HE Merkel N Posselt HG Staab D Sieder C Pharmacokinetics and Safety of an 8 Week Continuous Treatment With Once-Daily Versus Twice-Daily Inhalation of Tobramycin in Cystic Fibrosis Patients. J Antimicrob Chemother (2016) 71(3):711–7. 10.1093/jac/dkv399 26626719

[13] Burdette SD Limkemann AJ Slaughter JB Beam WB Markert RJ. Serum Concentrations of Aerosolized Tobramycin in Medical, Surgical, and Trauma Patients. Antimicrob Agents Chemother (2009) 53(10):4568. 10.1128/AAC.00490-09 19687242 PMC2764167

[14] van Velzen AJ Bos AC Touw DJ Tiddens HA Heijerman HG Janssens HM. Pharmacokinetics and Tolerability of Once Daily Double Dose Tobramycin Inhalation in Cystic Fibrosis Using Controlled and Conventional Nebulization. J Aerosol Med Pulm Drug Deliv (2016) 29(3):273–80. 10.1089/jamp.2015.1259 26716357

[15] Laporta R Ussetti P Carreño MC. Renal Toxicity Due to Inhaled Tobramycin in Lung Transplant Recipients. J Heart Lung Transpl (2006) 25(5):608. 10.1016/j.healun.2005.10.008 16678043

[16] Ahya VN Doyle AM Mendez JD Lipson DA Christie JD Blumberg EA Renal and Vestibular Toxicity Due to Inhaled Tobramycin in a Lung Transplant Recipient. J Heart Lung Transpl (2005) 24(7):932–5. 10.1016/j.healun.2004.05.008 15982625

[17] Mora CVM Borja Vargas N Iturbe Fernández D Tello Mena S Cifrián Martínez JM. Exacerbation of Chronic Renal Failure Because of Inhaled Tobramycin in a Lung Transplant Patient. Respir Med Case Rep (2022) 36:101584. 10.1016/j.rmcr.2022.101584 35127435 PMC8803646

[18] Schultheis JM Durham ME Kram SJ Kuhrt M Gilstrap DL Parish A Incidence and Associated Risk Factors for Systemic Drug Levels With Inhaled Aminoglycoside Therapy. J Antimicrob Chemother (2023) 78(2):450–6. 10.1093/jac/dkac412 36512376 PMC10169422

[19] Kim J Waldman G Marks CR Gandhi R Palafox J Neuringer I Detectable Serum Concentration With Prophylactic Inhaled Tobramycin in Lung Transplant Recipients. J Heart Lung Transplant (2022) 41(4):413. 10.1016/j.healun.2022.01.1040

[20] Mehta RL Kellum JA Shah SV Molitoris BA Ronco C Warnock DG Acute Kidney Injury Network: Report of an Initiative to Improve Outcomes in Acute Kidney Injury. Crit Care (2007) 11(2):R31. 10.1186/cc5713 17331245 PMC2206446

[21] Hubert D Leroy S Nove-Josserand R Murris-Espin M Mely L Dominique S Pharmacokinetics and Safety of Tobramycin Administered by the PARI eFlow Rapid Nebulizer in Cystic Fibrosis. J Cyst Fibros (2009) 8(5):332–7. 10.1016/j.jcf.2009.07.001 19651542

[22] Mayo Clinic Laboratories. Tobramycin, Random, Serum (2024). Available from: https://www.mayocliniclabs.com/test-catalog/overview/37065#Clinical-and-Interpretive (Accessed March 6, 2024).

[23] Droege CA Ernst NE Foertsch MJ Bradshaw PG Globke AE Gomaa D Assessment of Detectable Serum Tobramycin Concentrations in Patients Receiving Inhaled Tobramycin for Ventilator-Associated Pneumonia. Respir Care (2022) 67(1):16–23. 10.4187/respcare.09412 34815325

[24] Doricic J Greite R Vijayan V Immenschuh S Leffler A Ius F Kidney Injury After Lung Transplantation: Long-Term Mortality Predicted by Post-Operative Day-7 Serum Creatinine and Few Clinical Factors. PLoS One (2022) 17(3):e0265002. 10.1371/journal.pone.0265002 35245339 PMC8896732

