# Supplementary material for: Tobramycin Systemic Absorption in Lung Transplant Recipients Treated With Inhaled Tobramycin: A Cohort Study
Source: Transpl Int. 2024 Mar 28;37:12579. doi: 10.3389/ti.2024.12579 (PMC11007664; doi:10.3389/ti.2024.12579)
Supplement: Supplementary file 1 [file Table1.docx]

**Supplementary table 1.** Group 1 - Patients on invasive mechanical ventilation.

| **Patient** | **Age** | **Isolated bacteria** | **Type of infection** | **Time of inhaled Tobramycin (days) until first and second measure** | **First tobramycin trough serum concentration (µg/mL)** | **Second tobramycin trough serum concentration**  **(µg/mL)** | **Baseline creatinine prior to commencing nebulized tobramycin (mg/dL)** | **Acute Kidney Injury (peak of creatinine mg/dL)** | **Cause of stopping tobramycin**  **(days)** | **Systemic antibiotic during nebulized tobramycin** |
| --- | --- | --- | --- | --- | --- | --- | --- | --- | --- | --- |
| 1 | 47 (F) | MSSA | Tracheobronchitis | 3/6 | 0.43 | 1.61 | 0.57 | No | - | Cloxacillin |
| 2 | 64 (F) | *P. aeruginosa* and *Serratia. marcescens* | Donor bronchial aspirate positive and tracheobronchitis | 4/7 | 0.14 | 0.56 | 0.38 | No | - | None |
| 3 | 61 (F) | *Klebsiella pneumoniae* and *Chryseobacterium* spp. | Tracheobronchitis | 7/25 | 1.07 | 0.31 | 0.40 | No | - | None |
| 4 | 52 (M) | *S. marcescens* | Donor bronchial aspirate positive | 106/ND | 1.67 | - | 0.30 | No | - | None |
| 5 | 53 (F) | *S. marcescens* | Donor bronchial aspirate positive | 4/7 | 9.53 | 0.54 (3 days after stopping treatment with inhaled tobramycin) | 0.74 | Yes (1.25) | High trough concentration and acute kidney injury (4) | Meropenem |
| 6 | 61 (F) | MSSA and *K. pneumoniae* | Donor bronchial aspirate positive and tracheobronchitis | 2/6 | 3.4 | 9.84 | 0.80 | Not valuable because of CVVH immediately after transplant | High trough concentration (6) | Cloxacillin |
| 7 | 64 (M) | *P.  aeruginos*a and *S.  marcescens* | Tracheobronchitis | 4/19 | 1.48 | 0.97 | 0.78 | No |  | Amoxicillin-clavulanate + ceftazidime |
| 8 | 58 (M) | MSSA | Donor bronchial aspirate positive | 5/16 | 1.89 | 2.39 | 0.70 | No | High trough concentration (16) | Amoxicillin-clavulanate + ceftazidime |
| 9 | 60 (M) | MSSA | Donor bronchial aspirate positive | 9/26 | 1.92 | 0.43 | 0.70 | No | - | Meropenem |
| 10 | 40 (F) | *K. pneumoniae* | Donor bronchial aspirate positive | 4/ND | 5.01 | - | 0.97 | Yes (1.63) | High trough concentration and acute kidney injury (5) | Meropenem + levofloxacin |
| 11 | 50 (M) | MSSA | Donor bronchial aspirate positive | 13 /22 | 0.47 | 0.64 | 0.88 | No | - | None |
| 12 | 39 (F) | MSSA and *Haemophilus influenzae* | Donor bronchial aspirate positive | 1/ND | 5.98 | - | 0.70 | Yes (1.86) | Acute kidney injury (3) | Cloxacillin |
| 13* | 53 (F) | *Ochrobactrum anthropi* | Donor bronchial aspirate positive | 8/ND | 0.88 | - | 0.55 | No | - | None |
| 14 | 66 (M) | *K. pneumoniae* and *Corynebacterium amycolatum* | Tracheobronchitis | 15/28 | 3.81 | 6.65 | 0.75 | Yes (1.77) | High trough concentration and acute kidney injury (29) | Piperacillin-tazobactam |
| 15 | 68 (M) | *E. cloacae* and *S.  marcensens* | Tracheobronchitis | 5/48 | 1.78 (IMV) | <0.1 (without IMV) | 0.79 | Yes (2.36) | Acute kidney injury (334) | None |

Abbreviations: *C*CVVH: Continuous Veno-Venous Hemofiltration; F: female; IMV: Invasie Mechanical Ventilation; M: male; MSSA: Methicillin-Susceptible *Staphylococcus aureus;* MRSA: Methicillin-Resistant *Staphylococcus aureus;*
