# Supplementary material for: Tobramycin Systemic Absorption in Lung Transplant Recipients Treated With Inhaled Tobramycin: A Cohort Study
Source: Transpl Int. 2024 Mar 28;37:12579. doi: 10.3389/ti.2024.12579 (PMC11007664; doi:10.3389/ti.2024.12579)
Supplement: Supplementary file 2 [file Table2.docx]

**Supplementary table 2.** Group 2 – Patients without invasive mechanical ventilation.

| **Patient** | **Age** | **Isolated bacteria** | **Type of infection** | **Time of inhaled Tobramycin (days) until first and second measure** | **First tobramycin trough serum concentration (µg/mL)** | **Second tobramycin trough serum concentration**  **(µg/mL)** | **Baseline creatinine prior to commencing nebulized tobramycin (mg/dL)** | **Acute Kidney Injury (peak of creatinine mg/dL)** | **Cause of stopping tobramycin**  **(days)** | **Systemic antibiotic during nebulized tobramycin** |
| --- | --- | --- | --- | --- | --- | --- | --- | --- | --- | --- |
| 1 | 49 (M) | MSSA | Donor bronchial aspirate positive | 56 days/ND | 0.37 | - | 0.97 | No | - | None |
| 2 | 28 (F) | MSSA and *P.  aeruginosa* | Donor bronchial aspirate positive | 5/138 days | <0.1 | <0.1 | 0.78 | No | - | None |
| 3 | 64 (M) | MSSA | Donor bronchial aspirate positive and tracheobronchitis | 46 days/ND | 0.65 | - | 1,03 | No | - | None |
| 4 | 53 (M) | MSSA | Donor bronchial aspirate positive and pneumonia | 12/52 days | 0.11 | 0.33 | 0.64 | No | - | Cloxacillin |
| 5 | 52 (M) | *Bacillus cereus* | Tracheobronchitis | 8/21 days | 0.29 | <0.1 | 0.36 | No | - | None |
| 6 | 60 (M) | MSSA | Donor bronchial aspirate positive | 11 days /ND | 0.14 | - | 0.68 | No | - | None |
| 7 | 67 (M) | *K. oxytoca* and *K. pneumoniae* | Donor bronchial aspirate positive | 5 months/ND | 0.53 | - | 1.21 | Yes (1.73) | - Acute kidney injury (213) | None |
| 8 | 60 (M) | *K. oxytoca* | Pneumonia | 5.4/6 months | 2.8 | 0.55 | 1.65 | Yes (2.93) | Acute kidney injury (182) | None |
| 9 | 59 (F) | MSSA | Tracheobronchitis | 4.1 months/ND | <0.1 | - | 0.8 | No | - | None |
| 10* | 65 (M) | *MSSA and P.  aeruginosa* | Donor bronchial aspirate positive | 47.6 months/ND | 0.57 | - | 0.91 | No | - | None |
| 11 | 66 (M) | No isolation | Tracheobronchitis | 3.2 months/ND | 2.2 | - | 0.93 | Yes (1.66) | Acute kidney injury (105) | None |
| 12 | 62 (F) | MSSA | Donor bronchial aspirate positive | 2.4 months/ND | 2.11 | - | 0.77 | Yes (1.79) | Acute kidney injury (75) | None |
| 13# | 65 (M) | MSSA | Bronchial suture infection | 4.5 months/ND | <0.1 | - | 0.74 | No | - | None |
| 14*# | 66 (M) | MSSA | Tracheobronchitis | 64.5/69.5 months | <0.1 | <0.1 | 1.06 | No | - | None |
| 15* | 65 (F) | MSSA | Pneumonia | 9.3 months/ND | 0.15 | - | 0.4 | No | - | None |

Abbreviations: *C*CVVH: Continuous Veno-Venous Hemofiltration; F: female; IMV: invasive mechanical ventilation; M: male; MSSA: Methicillin-Sensitive *Staphylococcus aureus;* MRSA: Methicillin-Resistant *Staphylococcus aureus*

*These patients received inhaled tobramycin 300 mg every 24 hours.

#These three patients received single lung transplant
